# Serum choline in extremely preterm infants declines with increasing parenteral nutrition

**DOI:** 10.1007/s00394-020-02312-2

**Published:** 2020-06-25

**Authors:** Anders K. Nilsson, Anders Pedersen, Daniel Malmodin, Anna-My Lund, Gunnel Hellgren, Chatarina Löfqvist, Ingrid Hansen Pupp, Ann Hellström

**Affiliations:** 1 Section for Ophthalmology, Department of Clinical Neuroscience, Institute of Neuroscience and Physiology, Sahlgrenska Academy, University of Gothenburg, Gothenburg, Sweden; 2 Swedish NMR Centre, University of Gothenburg, Gothenburg, Sweden; 3 Department of Clinical Sciences Lund, Pediatrics, Lund University, Skane University Hospital, Lund, Sweden; 4 Institute of Biomedicine, Sahlgrenska Academy, University of Gothenburg, Gothenburg, Sweden; 5 Institute of Health and Care Sciences, Sahlgrenska Academy, University of Gothenburg, Gothenburg, Sweden

**Keywords:** Betaine, Enteral nutrition, Methionine, Human milk, Phosphatidylcholine, Proton nuclear magnetic resonance

## Abstract

**Purpose:**

Choline is an essential nutrient for fetal and infant growth and development. Parenteral nutrition used in neonatal care lack free choline but contain small amounts of lipid-bound choline in the form of phosphatidylcholine (PC). Here, we examined the longitudinal development of serum free choline and metabolically related compounds betaine and methionine in extremely preterm infants and how the concentrations were affected by the proportion of parenteral fluids the infants received during the first 28 postnatal days (PNDs).

**Methods:**

This prospective study included 87 infants born at gestational age (GA) < 28 weeks. Infant serum samples were collected PND 1, 7, 14, and 28, and at postmenstrual age (PMA) 32, 36, and 40 weeks. The serum concentrations of free choline, betaine, and methionine were determined by ^1^H NMR spectroscopy.

**Results:**

The median (25th–75th percentile) serum concentrations of free choline, betaine, and methionine were 33.7 (26.2–41.2), 71.2 (53.2–100.8), and 25.6 (16.4–35.3) µM, respectively, at PND 1. The choline concentration decreased rapidly between PND one and PND seven [18.4 (14.1–26.4) µM], and then increased over the next 90 days, though never reaching PND one levels. There was a negative correlation between a high intake of parenteral fluids and serum-free choline.

**Conclusion:**

Circulating free choline in extremely preterm infants is negatively affected by the proportion of parenteral fluids administered.

**Trial registration:**

ClinicalTrials.gov Identifier NCT02760472, April 29, 2016, retrospectively registered.

**Electronic supplementary material:**

The online version of this article (10.1007/s00394-020-02312-2) contains supplementary material, which is available to authorized users.

## Introduction

Choline is an essential nutrient for humans and plays crucial roles in fetal and infant development. Choline is a principal component in the biosynthesis of structural and signaling phospholipids, including sphingomyelins, phosphatidylcholines (PC) and lyso-PC [1]. Enzymatic oxidation of choline yields betaine, a molecule that serves as a methyl donor for methionine synthesis and integrates with folate metabolism and glutathione synthesis [2–4]. Choline may also form an ester with acetic acid to produce the neurotransmitter acetylcholine [5]. PC, and thereby also choline, can be synthesized endogenously through sequential methylation of the head-group of phosphatidylethanolamine (PE) in the hepatic PE-*N*-methyltransferase (PEMT) pathway [6]. However, this pathway is not sufficient to meet the body’s requirement for choline and it must also be provided through the diet [7]. Fetal demand for choline is high during intrauterine life, particularly during the third trimester of pregnancy when choline is needed for extensive cell expansion and membrane biosynthesis [8]. The fetus increases in total body weight and brain weight about five times from gestational week 24–40 [9]. This period corresponds to the time that extremely preterm infants (born < 28 weeks of pregnancy) spend in the neonatal intensive care unit (NICU), and they are largely dependent on an exogenous choline supply. There are indications that preterm infants receiving parenteral nutrition (PN) suffer from choline undernourishment with potential consequences to growth and development [10–13].

Evidence from animal studies shows that high choline intake during gestation improves offspring brain development and cognitive ability [14]. There are also some studies in humans to support this conclusion [reviewed in 15]. In adults, choline restriction results in hepatic abnormalities due to the inability to assembled and secreted lipoprotein particles, resulting in reduced triglyceride transport out of the liver [16–19]. It is not known if choline deficiency in preterm infants contributes to PN-associated liver disease.

At term delivery, the choline concentration is approximately threefold higher in umbilical cord blood than maternal blood [20, 21]. The cord blood concentration of free choline inversely correlates with gestational age (GA) at birth [22]. After birth, breast milk provides the infant with choline. The predominant choline-containing compounds in breast milk are phosphocholine, glycerophosphocholine, and PC, with free choline, lyso-PC, and sphingomyelin found in lower quantities [23]. The breast milk from mothers delivering prematurely has been reported to contain lower total choline than milk from mothers delivering at term [23]. Recent estimations of the adequate intake of choline for preterm infants found that a daily intake of up to 76 mg/kg may be necessary to support the rapid growth at 24–34 weeks PMA [24]. However, the median choline intake during the first month of life for very preterm infants has been reported to be substantially lower [12]. Choline intake is especially low during the first week of life when PN intake is high and breast milk intake low. PN currently used for preterm infants do not contain free choline. Parenteral lipid emulsions contain a small fraction of PC (~ 5% of total lipids), which is added to emulsify the triglycerides carrying the bulk fatty acids. PC in parenteral lipid emulsions contains approximately 1.1 mg choline per mL, which corresponds to 5.5–16.5 mg choline when supplied in the normal range of 1–3 g fat/kg/day. However, only a minor fraction of the PC in the lipid emulsions is converted to free choline [13, 18]. The choline in PN lipid emulsions does not provide the infant with sufficient amounts to fulfill adequate intake.

In this study, we examined the longitudinal serum levels of free choline, betaine, and methionine in extremely preterm infants. We hypothesized that a high intake of parenteral fluids would have a negative impact on circulating free choline.

## Methods

### Study population

This investigation is part of the Donna Mega Study (NCT 02,760,472), a randomized open-label controlled trial with the primary aim of determining the role of parenterally administered omega-3 LC-PUFAs in growth and disease development [25]. Infants born < 28 weeks GA in the NICU at Sahlgrenska University Hospital in Gothenburg, Sweden, were eligible for inclusion in the study. Exclusion criteria were major malformations. Recruitment occurred between April 2013 and September 2015. Of 138 eligible infants, 90 were enrolled in the original study, and sufficient serum sample volumes for NMR analysis were available from 87 of these infants and could be included in the present study.

### Nutritional management and collection of nutritional data

Infants were randomized to receive one of two parenteral lipid emulsions, Smoflipid [30% soybean oil, 30% medium-chain triglycerides, 25% olive oil, and 15% fish oil (Fresenius Kabi AB, Uppsala, Sweden)] or ClinOleic [80% olive oil and 20% soybean oil, (Baxter Medical AB, Kista, Sweden)]. As soon as possible after birth, a parenteral solution containing amino acids and glucose was administered. Unless contraindicated, this was followed by the addition of a lipid emulsion between 6 and 12 h after birth. The parenteral nutrition protocol has been described in detail elsewhere [25].

The local routine for enteral nutrition included initiation of minimal enteral feedings with human milk within 3 h after birth, with enteral volumes gradually increased thereafter. Infants were fed maternal milk supplemented with pasteurized donor milk if required. Donor milk was given until 34 weeks PMA, after which it was replaced with preterm formula. Individualized fortification based on breast milk analysis of energy and macronutrient content was practiced for both maternal and donor milk. The enteral nutrition protocol has been described in more detail elsewhere [26]. Data regarding nutritional intake were collected prospectively during the first 28 days of hospitalization. All parenteral and enteral fluids (including human milk), fortifiers, supplements, and transfusions of blood products were recorded daily. Potential nutrients from blood products were not included in the data analysis. A more detailed description regarding the collection of nutritional data in this cohort was reported previously [26].

### Blood collection and sample preparation

Venous blood samples were collected PND 1, 7, 14, and 28, and PMA 32, 36, and 40 weeks. Blood samples were kept at 4 °C for a minimum of 45 min and a maximum of 2 h before centrifugation and serum collection. Samples were stored at − 20 °C for up to 1 week before long-term storage at − 80 °C until analysis. All samples had been subjected to at least one, but less than five, freeze–thaw cycles prior to analysis. In cryo vials, 50 µl ultrapure water and 100 µl buffer (75 mM sodium phosphate pH 7.4, 2.232 mM TSP-d4, 0.1% sodium azide in 20% D_2_O) were added to 50 µl of serum and then transferred to 3 mm SampleJet NMR tube racks (Bruker BioSpin). All liquid handling was performed by a SamplePro L robot (Bruker BioSpin) equipped with two cooling stations set to 2 °C to keep the cold chain throughout sample preparation.

### NMR data acquisition and processing

NMR data were acquired on an Oxford 800 MHz magnet equipped with an Avance III HD console and 3 mm TCI cryoprobe (Bruker BioSpin). Samples were kept at 6 °C in the SampleJet before measurement at 25 °C. One-dimensional excitation sculpting with perfect echo pulse sequence (‘zgespe’) was used to acquire profiling data. With a sweep width of 20 ppm, 128 scans were acquired in 64 k points, using an acquisition time of 2.04 s and relaxation delay of 3 s. Data were zero-filled to 128 k and Fourier-transformed including 0.3 Hz exponential line-broadening. Spectra were phased and referenced to the TSP-d4 signal. All processing was performed in TopSpin 3.5 pl6 (Bruker BioSpin).

### Peak integration, annotation, and quantification

Processed data were imported into MatLab (Mathworks Inc.). Peaks were aligned with Interval correlation optimized shifting [icoshift, 27] and integrated into a linear baseline using in-house routines. Tentative annotations of peaks were made using ChenomX 8.3 (ChenomX Inc.) and the spectral data in the Human Metabolome Data Bank [28]. Choline, betaine, and methionine were quantified by comparing isolated metabolite signal integrals to a reference standard sample (Bruker BioSpin) of known concentration for which 1D NMR data were acquired using identical experimental parameters as the serum samples.

### Ethics

The Donna Mega trial was approved by the Regional Ethical Review Board, Gothenburg (Dnr 303-11). Informed signed consent was obtained for all participants from their parents or legal guardians.

### Statistical analysis and artwork

Statistical analyses were performed using IBM SPSS Statistics version 26 (IBM corporation, Armonk, NY, US). Two-sided *p* < 0.05 was considered significant. Concentrations are reported as median and 25th/75th percentiles. Non-parametric Spearman’s rank for raw data or parametric Pearson’s *r* for log-transformed data were used for correlation analyses. Wilcoxon signed-rank test and Mann–Whitney *U* test were applied for comparisons of related and unrelated groups, respectively. Both PND and PMA were tested as underlying time scales when analyzing changes in postnatal serum levels of metabolites. Concentration changes were found to be dependent on the postnatal age of the infant rather than PMA (data not shown); therefore, the PND at sample acquisition was used as the time scale in all analyses.

Enteral fluids (mL/kg body weight) included the mother’s own milk and donor breast milk. Parenteral fluids (mL/kg body weight) included glucose and amino acid solutions, lipid emulsions, and other non-nutritional fluids. Blood products were not included in parenteral fluids. For calculation of enteral and parenteral fluid and lipid intake, recorded intake from birth to PND seven, from PND 8 to 14, and from PND 22 to 28 were summarized, representing postnatal week 1, 2, and 4, respectively. The proportions of parenteral fluids during weeks 1, 2, and 4 were obtained by dividing by total fluids (enteral + parenteral) during the corresponding periods and multiplying by 100. When investigating the effect of parenteral fluids on infant serum metabolite concentrations, infants were categorized into two equal-sized groups according to whether they received more or less than the median of fluids as parenteral fluids (median = 74, 37, and 11% as parenteral fluids during the 1st, 2nd, and 4th week, respectively). In multivariable regression models, the proportion of parenteral fluids was used as a continuous variable.

For calculations of total choline intake, parenteral lipid emulsions were assumed to contain 1.1 mg lipid-bound choline per mL from the egg lecithin emulsifier, and milk to contain 13.7 mg total choline per 100 mL [12]. No distinction was made between mother’s own milk and donor milk in terms of choline content. The small contribution of choline from emulsifiers in administered fat-soluble vitamin emulsions was not considered.

Multivariable regression models were validated according to common practices: the error terms were normally distributed, residuals exhibited homoscedasticity, error terms were independent, no or few outliers were observed, and no multicollinearity was found. Models were similar when potential outliers were removed. Parameters used to describe models from the regression analyses were β, the slope of the specified independent variable in the model; 95% confidence interval (CI) for β; *p* value, the significance of the specified independent variable in the model; and model *R*^2^, the goodness-of-fit of the model.

Figures were created in R Studio using the ggplot2 package [29].

## Results

### Study cohort

Demographic data are reported in Table 1. Table 2 provides the infants’ nutritional intake during the 1st, 2nd, and 4th postnatal week.

**Table 1:** Demographic data of the study population (*n* = 87)

Variable	Value
Gestational age, weeks	25.4 (1.4)
Birth weight, g	780 (224)
Birth weight *z*-score	− 0.86 (1.36)
Boys	50 (57%)
Infants receiving Smoflipid	46 (52%)
Days to full enteral intake*	14.0 (10.8–19.3)
Death before 40 weeks PMA	9 (10%)

Data are given as mean (SD), *n* (%), or median (25th–75th percentiles)

*Defined as the first day of enteral intake of 150 ml/kg/day (*n* = 78, nine infants died before full enteral intake was reached)

**Table 2:** Nutritional intake during the first, second, and fourth postnatal week

Variable	Week 1	Week 2	Week 4
Enteral fluids, ml/kg	243 (151–351)	766 (539–1007)	1056 (668–1202)
Parenteral fluids, ml/kg	693 (522–832)	439 (168–698)	143 (0.0–515)
Percent parenteral fluids of total	74.0 (61.73–85.5)	37.2 (12.8–56.3)	11.4 (0.0–41.0)
Enteral lipids, g/kg	9.49 (5.52–13.1)	28.6 (21.1–37.6)	37.1 (25.0–45.6)
Parenteral lipids, g/kg	9.23 (7.77–11.1)	6.77 (1.33–10.7)	0.0 (0.0–4.18)
*n*	81	79	72

Values represent median and 75th–25th percentiles

### Postnatal serum concentration of choline and related metabolites

**Fig. 1:**
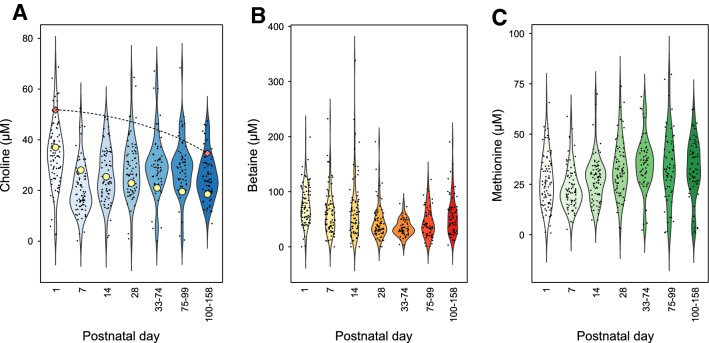
Violin plots showing serum concentrations of choline (**a**), betaine (**b**), and methionine (**c**) according to postnatal day. *n* = 85 (PNA d1), 79 (PNA d7), 81 (PNA d14), 72 (PNA d28), 81 (PNA d33-74), 52 (PNA d75-99), and 54 (PNA > 100). The dashed line in A indicate concentrations of free choline in cord plasma as reported by Bernhard et al. [22] based on median PMA 25.4 weeks at PNA day 1 and PMA 40 weeks at PNA day 100–158; circles show postnatal serum concentrations in full-term infants as reported by Ilcol et al. [30]

Highest serum concentrations of choline were seen at PND one, whereas the lowest concentrations were at PND seven, median (25th–75th percentile) 33.7 (26.2–41.2) vs. 18.4 (14.1–26.4) µM (Wilcoxon signed-rank test, *p* < 0.05, Fig. 1a). After PND seven, concentrations slowly increased. Serum betaine levels followed another pattern. The median betaine concentration was 71.2 (53.2–100.8) µM at PND one and then steadily declined to 34.2 (24.5–48.7) µM between PNDs 33–74 before increasing again (Fig. 1b). The serum betaine concentration was higher at PND one than later time points (Wilcoxon signed-rank test, *p* < 0.05). The median methionine concentration was 25.6 (16.4–35.3) µM at PND one, 21.0 (16.0–29.1) µM at PND seven, and then increased throughout the study period (Fig. 1c). The median methionine concentration was lower at PND one than PND 14 and later time points, but the concentration at PND seven was lower than any other time points (Wilcoxon signed-rank test, *p* < 0.05). Over the whole study period, concentrations of serum choline, methionine, and betaine correlated (*ρ* = 0.29–0.54 and *p* < 0.0001, Fig. S1). Correlations stratified by PND are reported in Table S1. Serum choline correlated with methionine throughout the study period (*ρ* = 0.262–0.557 and *p* < 0.05), and with betaine at all time points except PND 28 (*ρ* = 0.330–0.506 and *p* < 0.05, and *ρ* = 0.140 and *p* = 0.242, respectively). Betaine and methionine exhibited weak but significant correlations at PND 1, 14, and 28 (*ρ* = 0.272–0.455 and *p* < 0.05).

### Correlation between birth characteristics and metabolites

We found no significant correlation between GA at birth, birth weight, or birth weight *z*-score and the serum concentration of any of the analyzed metabolites at PND one.

### Influence of parenteral nutrition on serum choline concentration

**Fig. 2:**
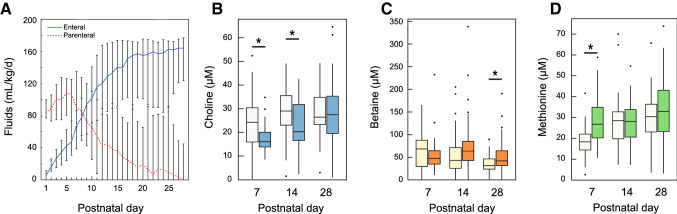
Postnatal changes in serum metabolites with the intake of enteral and parenteral fluids. **a** Median amount (ml/kg/d) of enteral (mother’s milk and donor milk, solid lines) and parenteral fluids (dashed lines) given during the infants’ first 28 postnatal days. Error bars indicate 25th and 75th percentiles. Box plots showing the concentration of (**b**) choline, (**c**) betaine, and (**d**) methionine if the infant was receiving less or more (darker boxes) than the median parenteral fluids. Whiskers indicate 25th and 75th percentiles. *p < 0.05, Mann–Whitney *U*

The decrease in serum choline concentration from birth to PND seven coincided with a high provision of parenteral fluids, peaking around PND five (Fig. 2a). Therefore, we tested whether the decrease in serum choline was influenced by the amount of parenteral fluids the infants were receiving. Infants receiving above the median parenteral fluids of total fluids (%) had significantly lower serum choline at PNDs seven and 14 [16.3 (13.9–21.7) µM vs. 24.3 (15.1–32.1) µM at PND seven, and 21.6 (16.6–33.2) µM vs. 29.0 (22.5–36.7) µM at PND 14, *p* = 0.006 and 0.010, respectively, Fig. 2b]. These infants also had significantly higher serum betaine levels at PND 28 [42.2 (32.0–64.1) µM vs. 31.6 (23.7–47.6) µM, *p* = 0.008, Fig. 2c] and methionine levels at PND 7 [24.4 (18.1–34.9) µM vs. 18.4 (13.9–22.3) µM, *p* < 0.001, Fig. 2d].

The influence of parenteral fluids on serum choline concentration was further tested by multivariable regression modeling (Table 3). After adjusting for choline at PND one and GA at birth, the model predicted that for every 1% increase in parenteral fluids as a proportion of total fluids during the first week of life, serum choline at PND seven would decrease by 0.33 µM (CI − 0.48–(− 0.17), model *p* = 0.0003).

**Table 3:** Multivariable regression analyses of the relationship between serum choline and parenteral nutrition

Dependent variable	*n*	Independent variables	*β*	95% confidence interval for β		*P*	*R*^2^
				Lower bound	Upper bound		
Choline PND seven (µM)	80					0.0003	0.22
		Choline PND 1 (µM)	0.165	0.00	0.33	0.049	
		Parenteral fluids week 1 (% of total fluids)	− 0.327	− 0.48	− 0.17	0.000	
		GA at birth (weeks)	− 0.677	− 2.31	0.96	0.412	

**Fig. 3:**
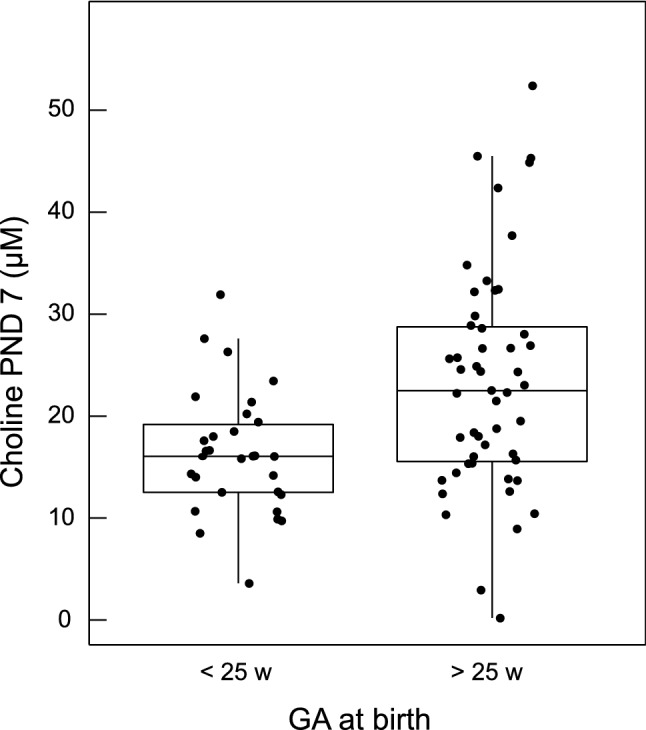
Choline concentration on postnatal day 7 based on gestational age at birth. Infants are grouped based on gestational age at birth < 25 weeks (*n* = 30) or > 25 weeks (*n* = 51). The serum concentration of choline was significantly higher in infants born after 25 weeks (Mann–Whitney *U*, *p* = 0.002). Whiskers indicate 25th and 75th percentiles

An indirect effect of parenteral fluids on serum choline was that more immature infants had lower choline on PND seven (Fig. 3). Infants born at GA < 25 weeks received a median 80% (73–90%) of total fluids as parenteral fluids during the first week of life, compared to 68% (55–81%) for infants born at GA > 25 weeks. Median serum choline in infants born at GA < 25 weeks was 16.0 µM (12.4–19.6) at PND 7, significantly lower than in infants born at GA > 25 weeks (22.5 µM [15.4–28.9], *p* = 0.002). There was no difference between groups in serum choline on PND one.

Infants born at GA < 25 weeks received median 11.3 (9.7–12.1) g parenteral lipids/kg during the first week, compared to 8.3 (7.1–9.8) g/kg for infants born at GA > 25 weeks (*p* < 0.0001). The parenteral lipids provided median 62 and 46 mg lipid-bound choline/kg during the first week for infants born at GA below and above 25 weeks, respectively. During the same time, milk was estimated to provide 26 and 39 mg total choline/kg for infants born at GA below and above 25 weeks, respectively. There was no difference between groups in calculated total choline intake.

## Discussion

Choline is crucial for fetal parenchymal growth and brain development. A growing body of evidence indicates that current nutritional practices do not satisfy the choline requirement for infants born extremely preterm. In the present study, we demonstrate in a cohort of extremely preterm infants that the free serum concentration of choline decreases as parenteral fluids are increased.

The concentration of free serum choline the first day of life was similar in this cohort as to what has been reported for free plasma choline levels in preterm infants [10, 22]. From PND one to PND seven, the serum choline concentration decreased by almost 50% from a median of 34–18 µM. The extent of this decrease was dependent on the fraction of parenteral fluids of total fluids the infant was receiving. Although this decrease in serum choline did not depend on GA at birth per se, the most immature infants (< 25 weeks GA at birth) showed significantly lower serum choline at PND seven due to high parenteral fluid intake. The decrease in serum choline that followed the administration of parenteral fluids appeared to be transient; once the infants increased their intake of breast milk, choline levels increased again. In agreement with this, free choline and other choline-containing metabolites in unpasteurized maternal breast milk are positively correlated with free choline in serum in full-term infants [30]. Notably, although free choline at day 7 was lower in infants born at GA < 25 weeks, the calculated intake of choline from parenteral lipids and milk during the first week of life was comparable to that of more mature infants. This is likely attributed to that milk and the PN contain different choline compounds, i.e., phosphocholine and glycerophosphocholine dominate in milk while parenteral lipids only contain PC that is poorly converted to free choline. Furthermore, more immature infants are believed to have higher choline requirements relating to metabolic rate, growth velocity, and plasma turnover, among other factors [12].

Our findings support previous calculations that choline intake is too low for maintaining adequate blood levels [12]; postnatal serum choline levels here were lower than reported for cord blood for the corresponding PMA (see Fig. 1a). Our data are also in line with a small study showing that preterm infants receiving ongoing total PN have lower plasma choline concentrations than infants only fed breast milk [10]. Other studies have reported that both children and adults who receive PN over extended periods have decreased circulating free choline and develop signs of hepatic dysfunction [16–18, 31, 32], confirming that endogenous synthesis through the hepatic PEMT pathway alone cannot fulfill the body’s requirement. Buchman et al. demonstrated that such conditions could be reversed by adding choline chloride to the PN [17].

In contrast to choline, the concentration of serum methionine increased with the proportion of parenteral fluids administered during the first postnatal week. This was not surprising, as methionine was included in the parenteral amino acids given to the infants (Vaminolac^®^, Fresenius Kabi). Nevertheless, serum methionine was lower on PND seven than PNDs one and 14. Notably, the choline and methionine correlations were lowest at PND seven, around the time when parenteral nutrition peaked.

Full-term infants experience a decrease in serum-free choline after birth [30]. However, this is a gradual decrease that coincides with an increase in phospholipid-bound choline, which is needed for membrane synthesis and tissue expansion [30]. For full-term infants, it takes approximately 6 months for choline levels to fall to half the concentration at PND one (compared to 7 days in the present cohort, see Fig. 1a) [30]. Thus, the rapid decrease in choline observed in extremely preterm infants, although transient, appears to be non-physiological and occurs when the choline requirement is high.

Betaine is a methyl donor for homocysteine in the synthesis of methionine and acts as an osmolyte that regulates cell volume, which is particularly important in the kidneys [33]. Approximately 2.6–7.3 mg/kg/day choline is irreversibly oxidized to betaine and excreted into the urine in preterm infants during the first postnatal week, independent of choline intake [34, 35]. The median daily intake of choline in our population during the first week of life was 12.3 mg/kg. Assuming 100% dietary choline absorption, approximately one-third of the consumed choline was oxidized to betaine and excreted, and as such was not available for further metabolism.

The excretion of betaine can be detected soon after birth and reaches a maximum at 2–3 months chronological age, then rapidly decreases over the next few months [34]. Peak excretion of betaine around 2–3 months chronological age coincides with minimum serum betaine concentration (~ 60 days) in the present study.

We did not find a significant influence of GA at birth on the choline concentration at PND one, as had been reported previously for choline in the cord blood plasma of preterm infants [22]. This may be explained by the current cohort representing a rather narrow range of GAs, from 22.7 to 27.9 weeks, compared to 24.3–40.7 weeks as reported by Bernhard et al. We did not detect any relationship between birth weight or the birth weight *z*-score and serum choline at PND 1. Cord blood choline and betaine levels are negatively associated with birth weight in full-term infants [20, 36]. The comparably small cohort size and the fact that serum samples were obtained on PND one rather than cord blood at birth may explain this discrepancy. However, we cannot exclude bodyweight relating to choline differently in preterm vs. full-term infants.

A limitation of the current study is that the actual intake of choline and related metabolites was not determined. When the intake of enteral fluids was calculated, we did not separate maternal and donor milk. The mother’s own preterm milk and donor milk appear to not differ in the concentration of water-soluble choline compounds (free choline, phosphocholine, and glycerophosphocholine) [37], whereas PC is known to decline during lactation and may be slightly higher in colostrum and transitional preterm milk than in mature donor milk [38]. Milk PC content is unlikely influenced by pasteurization [i.e., in donor milk, 39], but infant digestion and absorption of milk PC may be negatively affected by the inactivation of bile-salt stimulated lipase [40]. However, these quantitatively small differences in choline content between donor milk and the mother’s own milk and uptake in the infant are likely to have a limited impact on the infant’s choline status.

Although the American Society for Parenteral and Enteral Nutrition advocates that choline should be included in total PN [41], this has yet to be realized. Our data support previous conclusions that choline should be supplemented in extremely preterm infants as either an oral supplement or an integrated component of PN products.

## Electronic supplementary material

Below is the link to the electronic supplementary material.

Table S1. Spearman’s correlations between serum concentrations of choline, betaine, and methionine stratified by postnatal day. Figure S1. Associations between serum concentrations of choline, betaine, and methionine. Pearson's correlation coefficient (rho) and p-values from log-transformed data are indicated in the plots. Infant serum samples were collected between postnatal days 1 and 158. n=504 (PDF 741 kb)

## Data Availability

The data that support the findings of this study are available from the corresponding author upon reasonable request.
