# Supplementary material for: Serum choline in extremely preterm infants declines with increasing parenteral nutrition
Source: Eur J Nutr. 2020 Jun 25;60(2):1081–9. doi: 10.1007/s00394-020-02312-2 (PMC7900091; doi:10.1007/s00394-020-02312-2)
Supplement: Supplementary file 1 — Table S1. Spearman’s correlations between serum concentrations of choline, betaine, and methionine stratified by postnatal day. Figure S1. Associations between serum concentrations of choline, betaine, and methionine. Pearson's correlation coefficient (rho) and p-values from log-transformed data are indicated in the plots. Infant serum samples were collected between postnatal days 1 and 158. n=504 (PDF 741 kb) [file 394_2020_2312_MOESM1_ESM.pdf]

Serum choline in extremely preterm infants declines with parenteral nutrition

Anders K. Nilsson[1], Anders Pedersen[2], Daniel Malmodin[2], Anna-My Lund[3], Gunnel Hellgren[1,4], Chatarina Löfqvist[1,5], Ingrid Hansen Pupp[3], Ann Hellström[1]

1. Section for Ophthalmology, Department of Clinical Neuroscience, Institute of Neuroscience and Physiology, Sahlgrenska Academy, University of Gothenburg, Gothenburg, Sweden
2. Swedish NMR Centre, University of Gothenburg, Gothenburg, Sweden
3. Lund University, Skåne University Hospital, Department of Clinical Sciences Lund, Pediatrics, Lund, Sweden
4. Biomedicine, Sahlgrenska Academy, University of Gothenburg, Gothenburg, Sweden;
5. Institute of Health and Care Sciences, Sahlgrenska Academy, University of Gothenburg, Gothenburg, Sweden

Address correspondence to: Anders K. Nilsson, anders.k.nilsson@gu.se

Supplemental Table 1. Spearman's correlations between serum concentrations of choline, betaine, and methionine stratified by postnatal day.

	Postnatal day													
	1 (n=85)		7 (n=81)		14 (n=79)		28 (n=72)		33-74 (n=81)		75-99 (n=52)		100-158 (n=54)	
	Betaine	Methionine	Betaine	Methionine	Betaine	Methionine	Betaine	Methionine	Betaine	Methionine	Betaine	Methionine	Betaine	Methionine
Choline	0.501	0.450	0.506	0.262	0.386	0.463	0.140	0.557	0.330	0.501	0.343	0.434	0.451	0.502
	p<0.0001	p<0.0001	p<0.0001	p=0.0180	p=0.0004	p<0.0001	p=0.24	p<0.0001	p=0.0027	p<0.0001	p=0.0129	p=0.0013	p=0.0006	p=0.0001
Betaine		0.455		0.134		0.272		0.288		0.168		0.150		0.264
		p<0.0001		p=0.23		p=0.0154		p=0.0141		p=0.13		p=0.29		p=0.0536

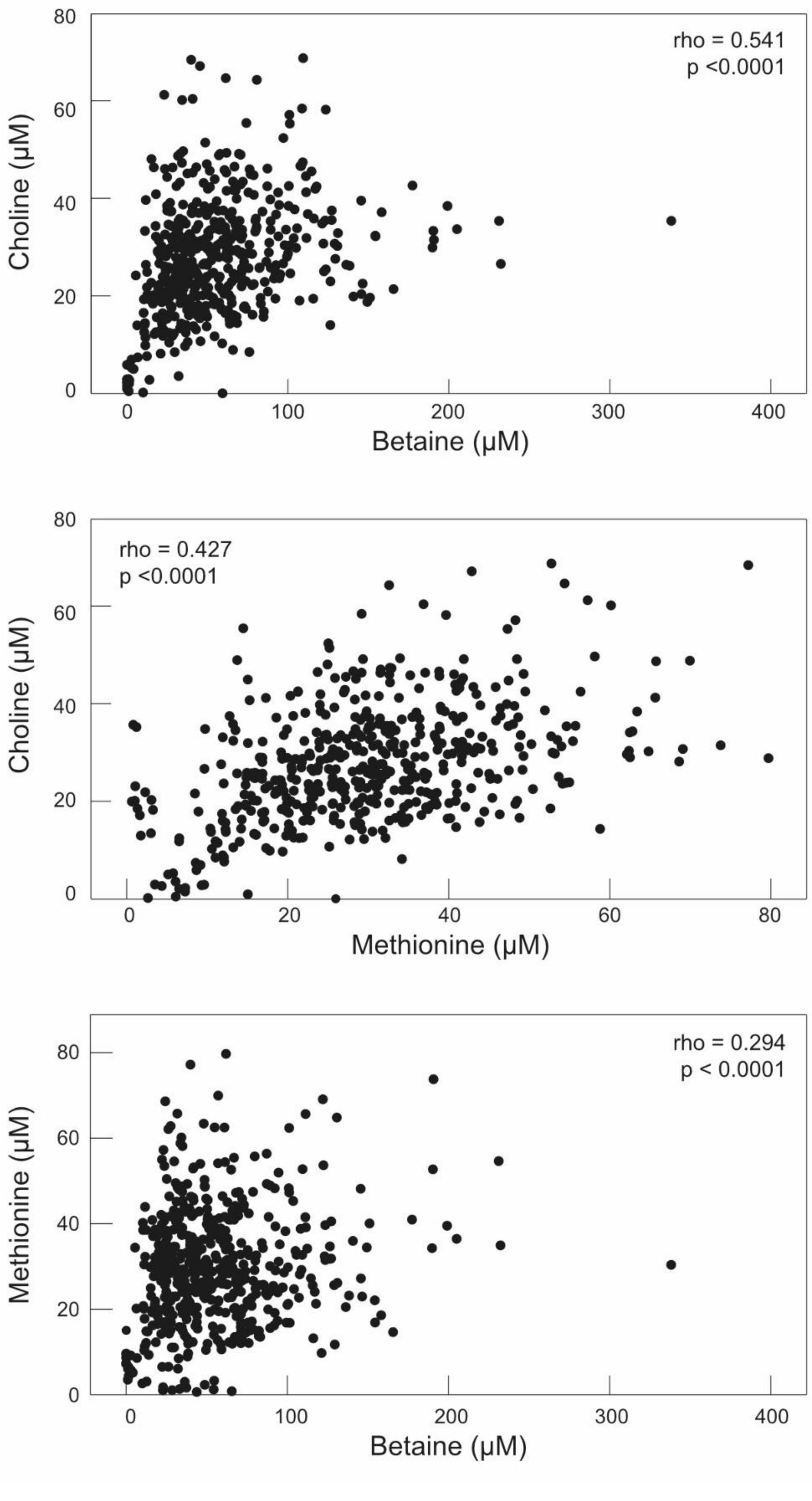

Supplemental Figure 1. Associations between serum concentrations of choline, betaine, and methionine. Pearson's correlation coefficient (rho) and p-values from log-transformed data are indicated in the plots. Infant serum samples were collected between postnatal days 1 and 158. n=504.
